# Fibrin formation and lysis in hyperglycaemia – molecular and cellular drivers

**DOI:** 10.1097/MOH.0000000000000932

**Published:** 2026-05-14

**Authors:** Julia S. Gauer

**Affiliations:** Discovery and Translational Science Department, Leeds Institute of Cardiovascular and Metabolic Medicine, University of Leeds, Leeds, UK

**Keywords:** cellular bioenergetics, fibrin formation, fibrinolysis, hyperglycaemia, thromboinflammation

## Abstract

**Purpose of review:**

Clot structure is a well established determinant of thrombosis risk. Hyperglycaemia alters clot architecture and function, producing denser, less permeable fibrin networks with reduced susceptibility to lysis. Although several mechanisms contributing to these effects have been reviewed previously, novel findings and growing evidence of cellular bioenergetics’ effect on clot structure have not yet been integrated into this context. This review presents new insights and discusses their relevance for future therapeutic development.

**Recent findings:**

Recent studies have identified novel glycation sites on fibrin(ogen), that contribute to altered microstructures and play important roles in FXIII cross-linking and plasmin cleavage. Increased level of fibrinolysis inhibitor PAI-1 was shown to precede the development of diabetes. Altered platelet and neutrophil bioenergetics in prediabetes and diabetes were shown to contribute to a procoagulant phenotype through increased oxidative stress and NETosis, respectively. Duration of hyperglycaemia and glycaemic control were shown to be key determinants influencing these cellular behaviours.

**Summary:**

Considering the interplay between the molecular and cellular drivers of hyperglycaemia-induced alterations in fibrin formation and lysis has important implications for identifying future therapeutic targets. Further research that encompasses hyperglycaemia duration and variations in glycaemic control may support the development of personalised strategies for thrombosis prevention.

## INTRODUCTION

Hyperglycaemia is a key feature of diabetes mellitus and metabolic syndrome [1]. Risk of thrombosis, including venous thromboembolism (VTE) and stroke, is higher in individuals with diabetes [2,3]. In a retrospective analysis, patients with pulmonary embolism and a comorbidity of metabolic syndrome were shown to have significantly increased rates of VTE recurrence [4]. Despite advances in therapy, reports indicate that deaths related to thrombosis account for up to 65% of deaths in individuals with diabetes [5]. Fibrin fibre structure is a well established risk factor for thrombosis [6,7,8] and potential predictor of causality and poor patient outcome [9,10]. In individuals with diabetes, denser and less porous fibrin networks with increased resistance to lysis have been reported [11,12]. This review will describe the hyperglycaemia-driven mechanisms affecting fibrin clot formation and lysis, explore the emerging evidence of cellular contributions, and describe potential implications for thrombosis risk management and therapeutic strategies. 

**Box 1:**
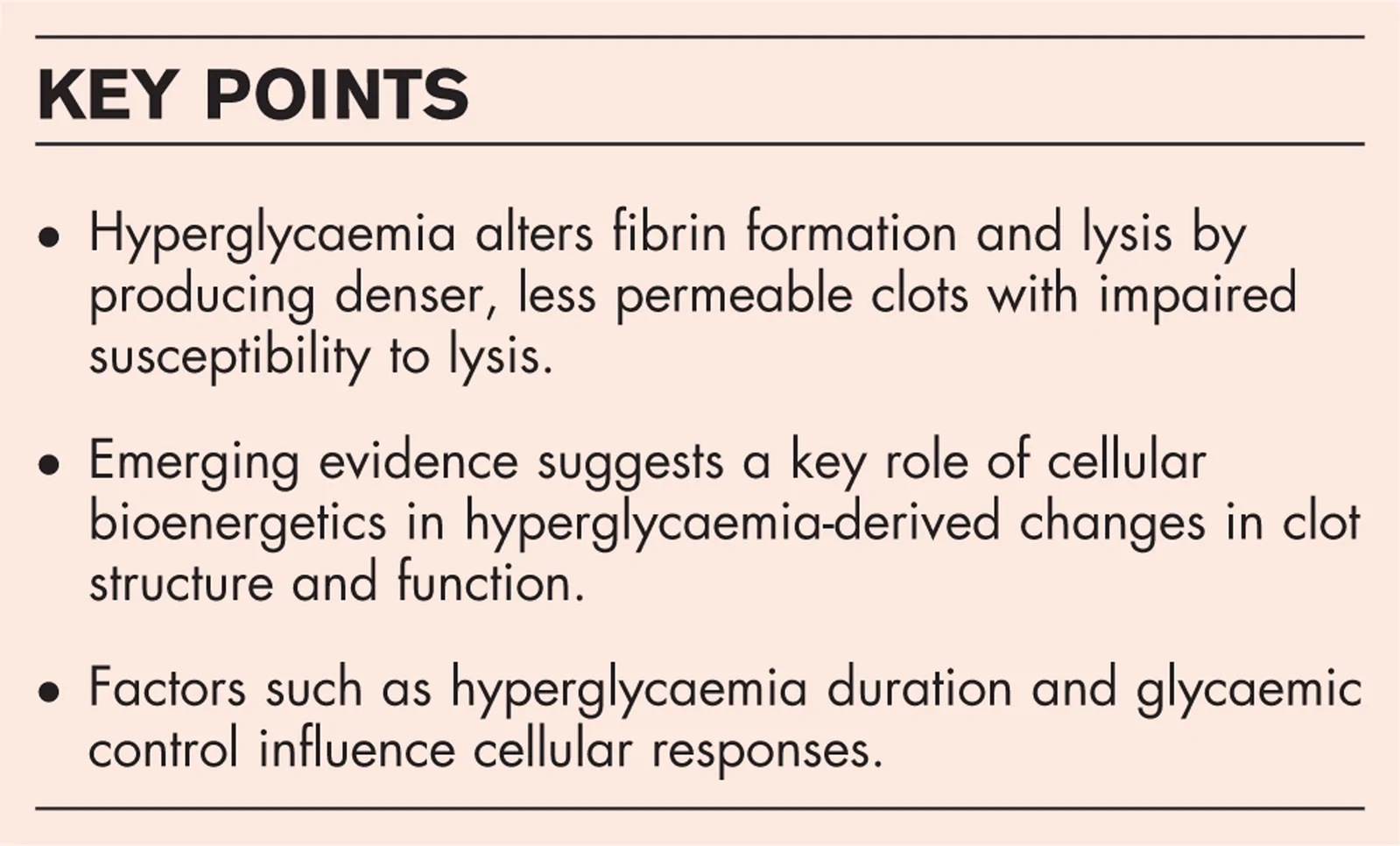
no caption available

## HYPERGLYCAEMIA AND FIBRIN FORMATION

Fibrinogen, a 340-kDa glycoprotein composed of two sets of Aα-, Bβ- and γ-chains, is the precursor to fibrin, an essential structural component of blood clots [6]. Several studies have shown altered fibrin(ogen) structure and function following exposure to hyperglycaemia [12–15]. In the following sub-sections, established mechanisms are presented alongside emerging data that elucidate how hyperglycaemia contributes to these alterations.

### Glycation

Glycation refers to the process in which glucose binds to proteins nonenzymatically. Increased levels of early-stage glycation compounds (ketoamines) and advanced glycation end-products (AGEs) are present in glycated fibrinogen samples [16]. Molecular docking simulations identified 477 hydrogen bonds (H-bonds) formed by the interaction of glucose with fibrinogen, and that the main residues involved in glucose-fibrinogen binding were aspartic acid, glycine and lysine [17]. In fact, an estimation using spectrophotometry showed reduced levels of free lysine residues in glycated fibrinogen samples [16,18]. In a previous small randomized control trial, incorporation of L-lysine supplementation to standard care for 3 months significantly decreased fasting blood glucose and fibrinogen levels in patients with diabetes, suggesting a potential clinical benefit of glycation inhibition [19]. More recently, in samples from 9 individuals with type 2 diabetes (T2D), glycation sites were found in all three chains of the fibrinogen molecule (10 on α-chain, 10 on β-chain and 6 on γ-chain) [20]. Visually, glycated fibrinogen has been attributed to distinct microstructures and increased aggregation in scanning electron microscopy (SEM) analysis [16,18]. In addition, transmission electron microscopy imaging of glycated fibrinogen showed increased irregularity and extended branching of fibres compared to nonglycated samples [21]. Similarly, fibrin clots formed with glycated fibrinogen showed increased fibrin fibre overlap and more rigid fibre microstructure in SEM and laser scanning confocal microscopy images, respectively [22].

### Fibrinogen cleavage and fibrin polymerisation

Hyperglycaemia has been shown to increase levels of pro-coagulation factors, including tissue factor and factor IX, and fibrinogen [23,24,25^▪^]. Elevated fibrinogen levels in diabetes, and possibly increased tissue factor and factor IX levels, have been suggested to be a consequence of increased hepatic synthesis in response to inflammatory cytokine activity driving a chronic inflammatory state [26]. Cleavage of fibrinopeptides by thrombin leads to the conversion of fibrinogen to fibrin monomers. Lateral aggregation and packing of fibrin oligomers (protofibrils) during fibrin polymerisation ultimately lead to the formation of the fibrin network [27]. Availability of thrombin directly influences rate of fibrin formation and also affects fibrin fibre thickness and the overall network density [28]. Increased peak thrombin concentration has been shown in patients with T2D [13]. Comparative studies also showed that fibrinogen from individuals with diabetes had higher clotting activity than fibrinogen from control group [12,29]. More recently, higher peak thrombin and endogenous thrombin potential (ETP) have been associated with the incidence of T2D [25^▪^]. In a 6-year follow-up study, higher peak thrombin concentration in individuals with T2D was associated with increased risk (>five-fold) of cardiovascular mortality [20]. Moreover, increased prothrombin and thrombin-antithrombin complexes, markers of thrombin formation, were observed in samples of acute coronary syndrome patients with hyperglycaemia or diabetes [30]. Another recent study on elderly individuals with peripheral arterial disease reported more than 40% increased peak thrombin concentration and thrombin generation rate in those with diabetes [31]. These findings suggest a potential role of thrombin levels in T2D cardiovascular complications.

### Cross-linking

Coagulation FXIII, a noncovalent heterotetramer (FXIII-A_2_B_2_) activated by thrombin, stabilises the forming fibrin network by cross-linking fibrin, thereby influencing clot architecture and its resistance to fibrinolysis [32]. FXIII cross-linking of fibrinogen α- and γ-chains occur through covalent bond formation between glutamine and lysine residues [33]. Glycation sites identified on fibrinogen α-chain in samples from individuals with TD2 are also reportedly important for FXIII cross-linking [20,33]. Previous reports suggest levels of FXIII B-subunit correlated with a surrogate model of insulin resistance in a healthy South Asian cohort, indicating a relationship between metabolic syndrome markers and coagulation factors in this population with a known high prevalence of insulin resistance [34]. Similarly, correlations were found between FXIII B-subunit and cardiovascular risk factors in individuals with T2D and their first-degree relatives [15,35], consistent with evidence that FXIII B-subunit levels are influenced by metabolic and inflammatory states rather than cardiovascular disease alone [36,37]. Although the aforementioned studies showed no correlations with FXIII A-subunit, absence of A-subunit in a murine model normalised glucose levels and protected animals from insulin resistance development following high fat diet [38]. These findings indicate that altered FXIII activity in hyperglycaemia contribute to a prothrombotic phenotype.

### Inflammation and oxidative stress

Hyperglycaemia-induced oxidative stress has been linked to the development of metabolic diseases and diabetes [39]. Exposure of fibrinogen to oxidative stress leads to the formation of denser fibrin clot networks [40–42]. In addition to affecting clot architecture, glycation and oxidation of fibrinogen has been linked with increased immunogenicity, potentially playing a role in the immune responses associated with diabetes [21]. Activation of inflammatory pathways through by binding of AGE to its receptor (RAGE), including nuclear factor-κB (NF-κB) activation, are associated with a pro-thrombotic phenotype [43,44]. As well as inducing reactive oxygen species formation, hyperglycaemia also increases expression of RAGE [45]. How oxidation of fibrinogen contributes to the development of thrombosis, including the potential protective effects of ‘low-dose’ oxidative stress, was the topic of a recent review [42]. Given the evidence that oxidative stress contributes towards increased thrombosis risk and heightened immunogenicity, antioxidant therapy may offer important therapeutic benefits in diabetes.

## HYPERGLYCAEMIA AND FIBRINOLYSIS

Previous studies indicate that hyperglycaemia and T2D impair fibrinolysis, a topic that has been the focus of several reviews [46–48]. Elevated oxidative stress, decreased affinity of plasminogen and tissue plasminogen activator (tPA) to fibrinogen, and the formation of denser, less permeable clot networks in T2D contribute to the slower lysis rates observed [12,49,50]. The following sub-sections describe emerging insights into how glycation and fibrinolysis inhibitors contribute to hypofibrinolytic effects.

### Glycation

Compared to nondiabetic controls, plasminogen extracted from samples of individuals with poorly controlled type 1 diabetes (T1D) had a higher percentage of glycation and showed decreased cleavage by plasmin [51]. This caused impaired fibrinolytic potential, which was partially recovered by improvement in glycaemic control [51]. More recently, a glycation site identified on α_2_-antiplasmin, a key inhibitor of fibrinolysis, has been shown to play a role in its cleavage by plasmin [52]. Another glycation site on fibrinogen α-chain in samples from individuals with TD2 was found to be involved in plasmin cleavage [20]. Overall, these data indicate that hyperglycaemia-associated glycation affects key fibrinolytic proteins, ultimately compromising fibrinolysis efficiency.

### Fibrinolysis inhibitors

Increased levels of plasminogen activator inhibitor (PAI)-1 in individuals with T2D have been reported in the late 1980s [53], sparking interest in the association between PAI-1 and diabetes. A multicentre follow-up study showed that increased level of PAI-1 from baseline was associated with hyperglycaemia and development of T2D [54]. Similarly, a recent study concluded that increased PAI-1 levels from baseline precede the development of T2D [55^▪^]. In nondiabetic individuals, a positive correlation between levels of serum AGE and PAI-1 was reported, suggesting a potential link between glycation and fibrinolysis ability [56]. Increased PAI-1 and antithrombin levels were also reported in a recent retrospective cross-sectional study including 250 women with gestational diabetes [57]. Although there are limited reports of increased PAI-1 levels in T1D [58], there is likely an effect of hyperglycaemia in the presence of insulin resistance [59]. Furthermore, a study in T1D mice showed that pharmacological inhibition of PAI-1 (with PAI-039) improved skeletal muscle regenerative capacity [60]. Nevertheless, a study on T1D individuals reported decreased PAI-1 activity and clot lysis time compared to controls, despite elevated levels of antiplasmin incorporation into the clot [61]. Another study reported less pronounced effects on clot lysis time in T1D and maturity onset diabetes of the young (MODY) compared to T2D [62]. These discrepancies underscore the therapeutic potential of targeting fibrinolysis inhibitors in hyperglycaemia whilst also emphasising the need for further investigation into the roles of hyperglycaemia duration and diabetes type.

## CELLULAR CONTRIBUTIONS

Hyperglycaemia is associated with platelet hyperreactivity and increased neutrophil extracellular trap formation (NETosis) [63–65], contributing to heightened coagulability and impaired wound healing. The potential of modulating platelet-neutrophil interactions to reduce thromboinflammation in the context of diabetes was discussed in a previous review [66]. The following sub-sections outline recent insights into the cellular mechanisms underlying the pro-thrombotic state associated with hyperglycaemia.

### Platelets

Activated platelets promote fibrin formation by supporting thrombin generation and regulate fibrinolysis through secretion of plasminogen and PAI-1, promoting either a pro or antifibrinolytic state depending on haemostatic stage [67,68]. A recent study using samples from individuals with prediabetes found elevated platelet activation and increased mitochondrial oxidant load, along with enhanced ex-vivo thrombus formation, compared with controls [69^▪^]. The same effects were observed regardless of age [69^▪^]. The authors propose that targeting mitochondrial oxidation-driven platelet activation could help alleviate the prothrombotic state associated with prediabetes [69^▪^]. Supporting this, an in-vitro study demonstrated that dietary antioxidants ameliorated acute hyperglycaemia-induced alterations in platelet bioenergetics [70]. Studies have also reported increased levels of platelet-derived microparticles in T1D and T2D [71,72]. Moreover, in hyperglycaemic T2D individuals, PAR-4 was identified as the mediator of platelet-derived microparticles release, which correlated with glycated haemoglobin (HbA1c) and the inflammation marker interleukin-6 (IL-6) [72]. Data from this study also showed higher MP release in samples from individuals with poor glycaemic control compared to good glycaemic control [72]. In T1D individuals, elevated HbA1c was also associated with higher counts of procoagulant platelet microparticles [71]. These observations highlight glycaemic control as important considerations for study design and data interpretation.

### Neutrophils and neutrophil extracellular traps

Neutrophils have been shown to promote fibrin formation by releasing neutrophil extracellular traps (NETs), which contribute to the formation of a denser and more lysis-resistant fibrin clot structure [73,74]. Increased levels of NETs and NETosis in T2D have been previously reported and associated with increased pro-inflammatory cytokines but not glycaemic control [65,75]. Nevertheless, a recent study reported increased basal NETosis levels in individuals with uncontrolled versus controlled diabetes [76]. Transcriptome analysis in the same study found upregulation of genes involved in glycolysis in diabetes and in-vitro hyperglycaemia-exposed neutrophils, suggesting a potential metabolic priming effect of hyperglycaemia [76]. In fact, glycolytic pathway inhibition reduced hyperglycaemia-induced NET formation [76]. Another study using plasma samples from individuals with T1D and T2D supplemented with NETs from healthy volunteers’ neutrophils found that addition of NETs increased clot stiffness and impaired fibrinolysis when compared to nondiabetes control samples [77]. Taken together, these findings suggest that targeting NET formation in the context of hyperglycaemia may offer therapeutic benefits by reducing inflammation and ameliorating pro-thrombotic clot phenotype.

### Endothelial dysfunction

Endothelial dysfunction is associated with the release of pro-thrombotic mediators, including tissue factor and PAI-1, leading to increased fibrin formation and impaired fibrinolysis [78,79]. Hyperglycaemia is known to contribute to endothelial dysfunction, with several reviews discussing the mechanisms at play and potential therapeutic implications [80–82]. Targeting hyperglycaemia-induced senescence-associated secreted protein (SASP) factors, such as PAI-1 and IL-6, may provide important benefits in preventing the development thromboinflammatory phenotype [83,84]. A recent study reported partial recovery of endothelial function upon return to normoglycaemia, measured by AGE accumulation, following acute hyperglycaemia in rats with streptozotocin-induced diabetes [85]. These findings suggest the existence of a transitional phase that may precede the development of irreversible dysfunction under chronic-hyperglycaemia conditions [85].

## TRANSLATIONAL AND CLINICAL IMPLICATIONS

**FIGURE 1:**
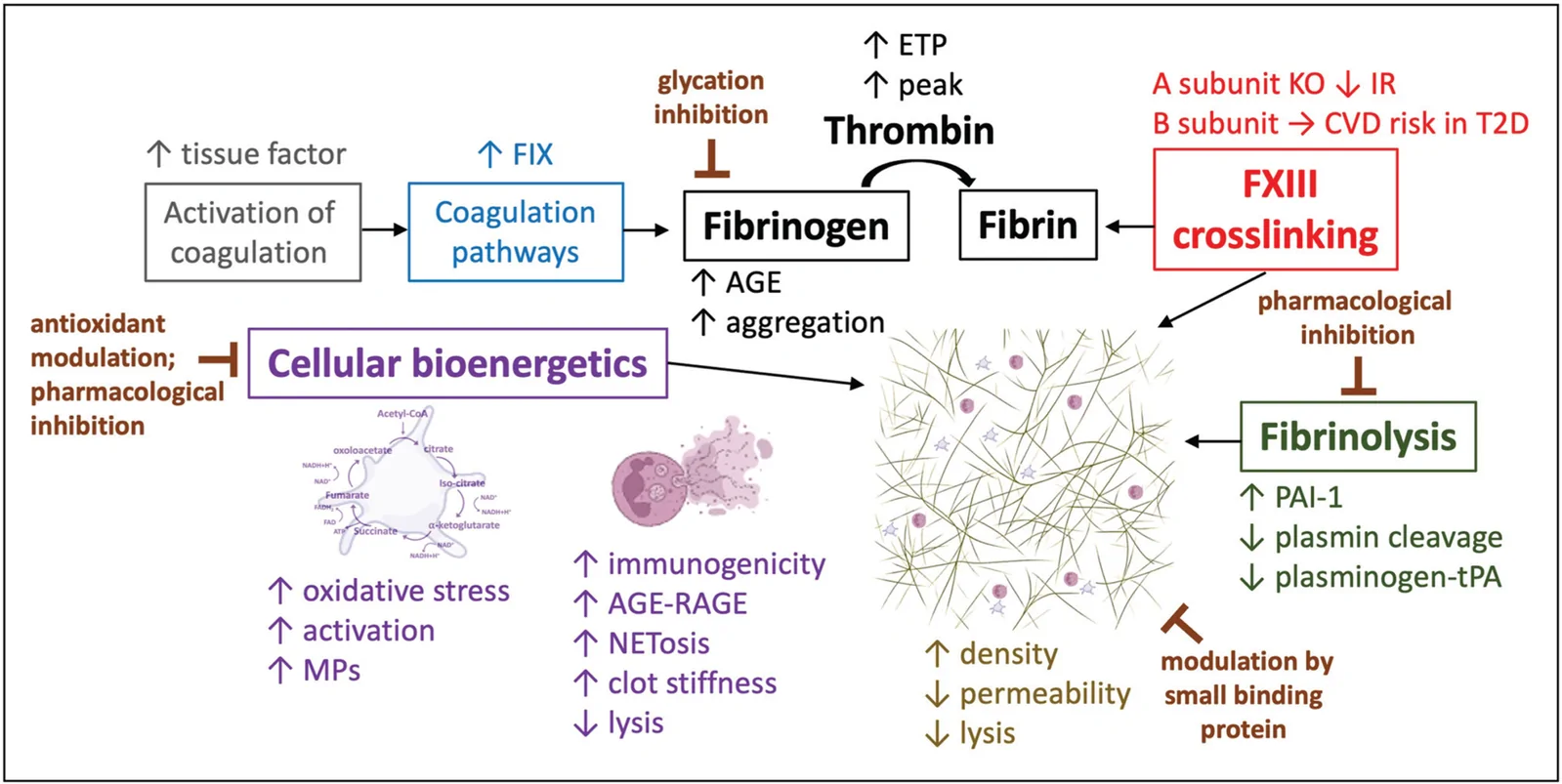
The effects of hyperglycaemia on fibrin formation and lysis. This figure describes the mechanisms that contribute to a prothrombotic and hypofibrinolytic phenotype in hyperglycaemia. Targets of potential therapeutic relevance are shown in brown. ↑ represents an increase; ↓ represents a decrease; → represents a correlation.

The findings discussed above are summarised in Table 1. Together, they highlight several potential mechanisms that warrant further investigation as therapeutic targets for mitigating the effects of hyperglycaemia on fibrin formation and lysis, as illustrated in Figure 1. For instance, ameliorating fibrinolytic potential by inhibiting PAI-1 may contribute to an effective preventive strategy for the development of T2D and complications of T1D-derived muscle regeneration impairment [55^▪^,^60^]. Of note, Metformin has been shown to decrease PAI-1 in individuals with T2D and insulin-resistance, indicating benefits for improving prothrombotic phenotype that extend beyond its effect on glycaemic control [46,86]. Although pharmacological PAI-1 inhibitor PAI-039 (tiplaxtinin) did not show efficacy in humans, more recent clinical trial with TM5614 report promising effects on malignant melanoma [87,88]. Further research is required to determine its potential effect on preventing and reducing thrombosis in the context of hyperglycaemia. Furthermore, due to its association with incidence of T2D [15,25^▪^], measures of thrombin and FXIII B-subunit levels have the potential to aid in risk stratification for diabetes development and cardiovascular disease complications.

**Table 1:** Summary of hyperglycaemia-induced effects of fibrin formation and lysis

HYPERGLYCAEMIA-INDUCED EFFECTS			
	Sample type	Reported effect	Reference
*Fibrin formation*			
*Glycation*	d-ribose glycated fibrinogen	↑ ketoamines & AGE	[16]
	Molecular docking simulations of glucose-fibrinogen binding	477 H-bonds; main residues involved = aspartic acid, glycine and lysine	[17]
	d-ribose and MGO glycated fibrinogen	↓ free lysine residues	[16,18]
	T2D	↓ FBG and fibrinogen post 3-month L-lysine supplementation + standard care.	[19]
	Fibrinogen from T2D	26 glycation sites: 10 on α-chain, 10 on β-chain & 6 on γ-chain	[20]
	d-ribose, MGO & glucose glycated fibrinogen	↑ aggregation & altered microstructure ↑ irregularity & extended branching ↑ fibrin fibre overlap	[16,18,22] [21] [22]
*Cleavage & polymerisation*	T2D	↑ tissue factor, FIX & fibrinogen	[23,24,25^▪^]
	T2D	↑ peak thrombin	[13]
	T2D	↑ clotting activity	[12]
	T2D	↑ peak thrombin & ETP → with incidence of T2D	[25^▪^]
	T2D	↑ peak thrombin → with CVD mortality	[20]
	Hyperglycaemia or T2D + ACS	↑ prothrombin & TAT	[30]
	T2D + PAD	↑ peak thrombin	[31]
*Cross-linking*	Fibrinogen from T2D	α-chain glycation site important for FXIII cross-linking	[20,33]
	Healthy SA individuals	FXIII B-subunit → IR	[34]
	T2D & first-degree relatives	↑ FXIII B-subunit	[15,35]
	Murine FXIII A-subunit KO	Normalised glycaemia & IR protection	[38]
*Inflammation & oxidative stress*	HOCl oxidised fibrinogen	↑ clot density	[40]
	MGO glycated fibrinogen	↑ immunogenicity	[21]
	T2D	AGE-RAGE activation of inflammatory pathways	[43,44]
	MGO treated HAECs	↑ ROS and RAGE expression	[45]
*Fibrinolysis*			
*Glycation*	T1D	↑ glycation & ↓ plasmin cleavage	[51]
	β-d-glucose glycated α_2_-antiplasmin	↓ plasmin cleavage	[52]
	T2D	α-chain glycation involved in plasmin cleavage	[20]
*Inhibitors*	T2D	↑ PAI-1 → hyperglycaemia & T2D development	[54,55^▪^]
	Healthy individuals	AGE → PAI-1	[56]
	GD	↑ PAI-1 & antithrombin	[57]
	Murine T1D	↓ PAI-1 improved muscle regenerative repair	[60]
	T1D	↓ PAI-1 & clot lysis; ↑ antiplasmin incorporation into clot	[61]
	T1D, T2D, MODY	Less pronounced change in clot lysis in T1D & MODY	[62]
*Cellular contributions*			
*Platelets*	Prediabetes	↑ activation, mitochondrial oxidant load & thrombus formation	[69^▪^]
	T1D & T2D	↑ MPs	[71,72]
	T1D	↑ HbA1c → ↑ MPs	[71]
*Neutrophils & NETS*	T2D	↑ NETs & NETosis	[65,75]
	Diabetes (unspecified)	↑ basal NETosis in uncontrolled vs. controlled diabetes ↑ glycolysis genes ↓ NETosis with pharmacological inhibition of ACLY & HATs	[76]
	T1D & T2D + healthy volunteers’ NETs	↑ fibrinolysis & clot stiffness	[77]
*Endothelial dysfunction*	Rats with STZ-induced diabetes	Recovery of endothelial function upon return to normoglycemia	[85]

↑ represents an increase, ↓ represents a decrease, → represents a correlation. ACLY, adenosine 5΄-triphosphate-citrate lyase; ACS, acute coronary syndrome; AGE, advanced glycation end-products; CVD, cardiovascular disease; ETP, endogenous thrombin potential; FIX, factor IX; FXIII, factor XIII; GD, gestational diabetes; HAECs, Human Aortic Endothelial Cells; HATs, histone acetyltransferases; HbA1c, glycated haemoglobin; H-bonds, hydrogen bonds; HOCl, Hypochlorous acid; IR, insulin resistance; KO, knock-out; MGO, methylglyoxal; MODY, Maturity-onset diabetes of the young; MPs, microparticles; NETosis, neutrophil extracellular trap formation; NETs, neutrophil extracellular traps; PAD, peripheral arterial disease; PAI-1, plasminogen activator inhibitor 1; RAGE, receptor for advanced glycation end-products; ROS, reactive oxygen species; SA, South Asian; STZ, streptozotocin; T1D, type 1 diabetes;

T2D, type 2 diabetes; TAT, thrombin-antithrombin complexes.

In addition, recent findings into the pivotal contributions of cellular bioenergetics to clot structure and function point to further promising therapeutic avenues for future research. For example, targeting mitochondrial-derived oxidative stress may diminish prothrombotic platelet activity in individuals with or at risk of developing diabetes [69^▪^]. In this context, dietary changes and antioxidant supplementation, alongside pharmacological treatments such as metformin and sodium-glucose co-transporter-2 (SGTL2) inhibitors, which have also been reported to decrease oxidative stress [89,90], could offer meaningful preventive benefits [66,91]. Dietary modifications may also contribute to decreased AGE accumulation [92]. Potential strategies to prevent the development of a thromboinflammatory phenotype include inhibiting excessive NETosis, reducing platelet-neutrophil aggregates, and limiting the release of pro-inflammatory and antifibrinolytic SASP factors [66,82,93]. Antiplatelet, anti-inflammatory and antioxidant effects have also been associated with glucagon-like peptide 1 receptor agonists (GLP-1RA) [94,95]. With the growing use of these agents globally, further research is required to decipher their effects on fibrin structure and function.

Glycation inhibition, through competition of L-lysine for glucose, for instance, represents another potential avenue for investigation [19]. Nevertheless, lysine analogues such as tranexamic acid (TXA), are widely used as fibrinolysis inhibitors [96]. Therefore, understanding how lysine supplementation may be used to modulate glycation without impairing fibrinolytic activity could be of therapeutic interest. Disrupting abnormal fibrin networks using small protein binders also represents a promising avenue for future research [97,98]. Additional key considerations for future studies include the duration of hyperglycaemia, the degree of glycaemic control, and potential sex-specific differences.

## CONCLUSION

Hyperglycaemia alters clot structure and function leading to the development of a prothrombotic and hypofibrinolytic clot phenotype. Growing research highlights the interconnected roles of blood cells with both coagulation and fibrinolysis systems in hyperglycaemia that have the potential to be explored as novel therapeutic targets. Future research that incorporates different disease types, comorbidities, and variations in glycaemic control is required to enhance risk prediction scores and improve thrombosis prevention. Such approaches may ultimately support the development of personalised strategies for thrombosis risk management in the context of hyperglycaemia.

## Acknowledgements

*None.*

### Financial support and sponsorship

*J.S.G. is supported in part by the National Institute for Health and Care Research (NIHR) Leeds Biomedical Research Centre. The views expressed are those of the author and not necessarily those of the NHS, the NIHR or the Department of Health and Social Care.*

### Conflicts of interest

*None.*

## REFERENCES AND RECOMMENDED READING

Papers of particular interest, published within the annual period of review, have been highlighted as:

▪ of special interest
▪▪ of outstanding interest
